# Depigmentation Therapy of Refractory Facial Vitiligo Using 532‐Nm Picosecond Laser: Two Case Reports and Literature Review

**DOI:** 10.1111/jocd.70604

**Published:** 2025-12-09

**Authors:** Xinxin Li, Xuhai Yuan, Juan Du, Xiaolan Ding, Fang Wang

**Affiliations:** ^1^ Department of Dermatology Peking University People's Hospital Beijing China

**Keywords:** 532‐nm picosecond, depigmentation therapy, laser therapy, vitiligo

## Abstract

**Background:**

Vitiligo is a common depigmenting disorder resulting from the loss of functional melanocytes. In certain refractory cases, conventional therapies fail to achieve complete repigmentation, leaving residual pigmented macules that can be cosmetically undesirable.

**Aims:**

To assess the efficacy and safety of 532‐nm picosecond laser combined with hydroquinone cream for treating residual pigmentation in vitiligo.

**Patients:**

We report two patients with stable generalized vitiligo who received 532‐nm picosecond laser treatment for residual pigmented macules. Patients subsequently applied 2% hydroquinone cream for 1–2 months. Clinical outcomes and adverse events were recorded. Written informed consent was obtained from both patients.

**Results:**

Marked reduction in residual pigmentation was observed in both patients, with cosmetically favorable outcomes. Mild erythema and burning sensation occurred immediately after treatment and resolved within 1 week.

**Conclusions:**

The 532‐nm picosecond laser, followed by topical hydroquinone, appears to be an effective and well‐tolerated approach for managing residual pigmentation in vitiligo. This reverse depigmentation strategy may serve as a valuable adjunct to enhance skin tone uniformity in selected patients.

## 1 Introduction

Vitiligo, an autoimmune‐mediated cutaneous disorder characterized by progressive epidermal melanocyte loss, presents as circumscribed or disseminated depigmented macules, imposing substantial psychosocial burden and social stigmatization. In refractory vitiligo cases unresponsive to pharmacological, phototherapeutic, or surgical interventions, residual normally pigmented patches may also induce psychosocial distress due to chromatic contrast. Therapeutic depigmentation protocols constitute a clinically validated strategy for this demographic subset, which directly targets residual dermal pigmentation foci characterized by histologically intact melanocytes with preserved melanosomes.

Current depigmentation modalities primarily involve chemical agents, cryotherapy, and laser‐based systems. Among laser approaches, various devices with different pulse durations and wavelengths are used for melanin targeting, among which the 532 nm picosecond devices show strong melanin absorption, especially in epidermal pigment. Picosecond pulse limits thermal diffusion and enhances photomechanical effects, thereby achieving more efficient pigment fragmentation than conventional nanosecond Q‐switched systems. Herein, we report two cases in which a 532‐nm picosecond laser was employed to address residual pigmented macules in Chinese patients with stable generalized vitiligo.

## 2 Case Reports

### 2.1 Case 1

**FIGURE 1:**
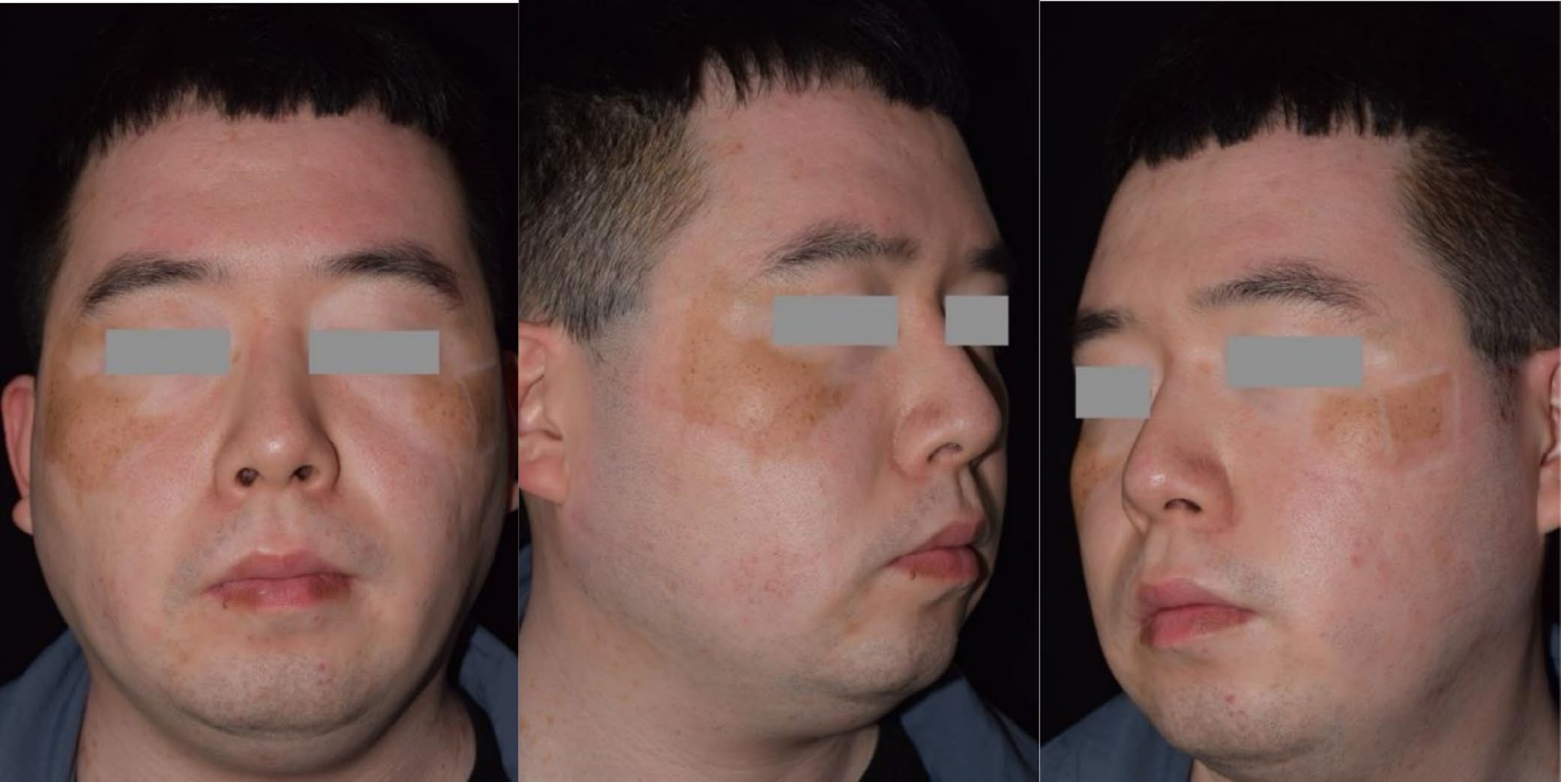
Pre‐treatment image of Patient 1, showing residual normal skin on both cheeks, contrasting with surrounding depigmented areas.

A 27‐year‐old male with an 11‐year history of generalized vitiligo, stabilized for 1 year, sought depigmentation treatment due to residual normal skin, particularly on the face, which caused significant cosmetic concern. Physical examination revealed well‐demarcated residual normal skin on both cheeks, contrasting with extensive depigmentation affecting approximately 99% of the body surface area (BSA). Scattered islands of repigmentation were also noted on the face (Figure 1). Additionally, partial leukotrichia was observed in the leg, while the scalp, eyebrows, and other body hair remained unaffected.

**FIGURE 2:**
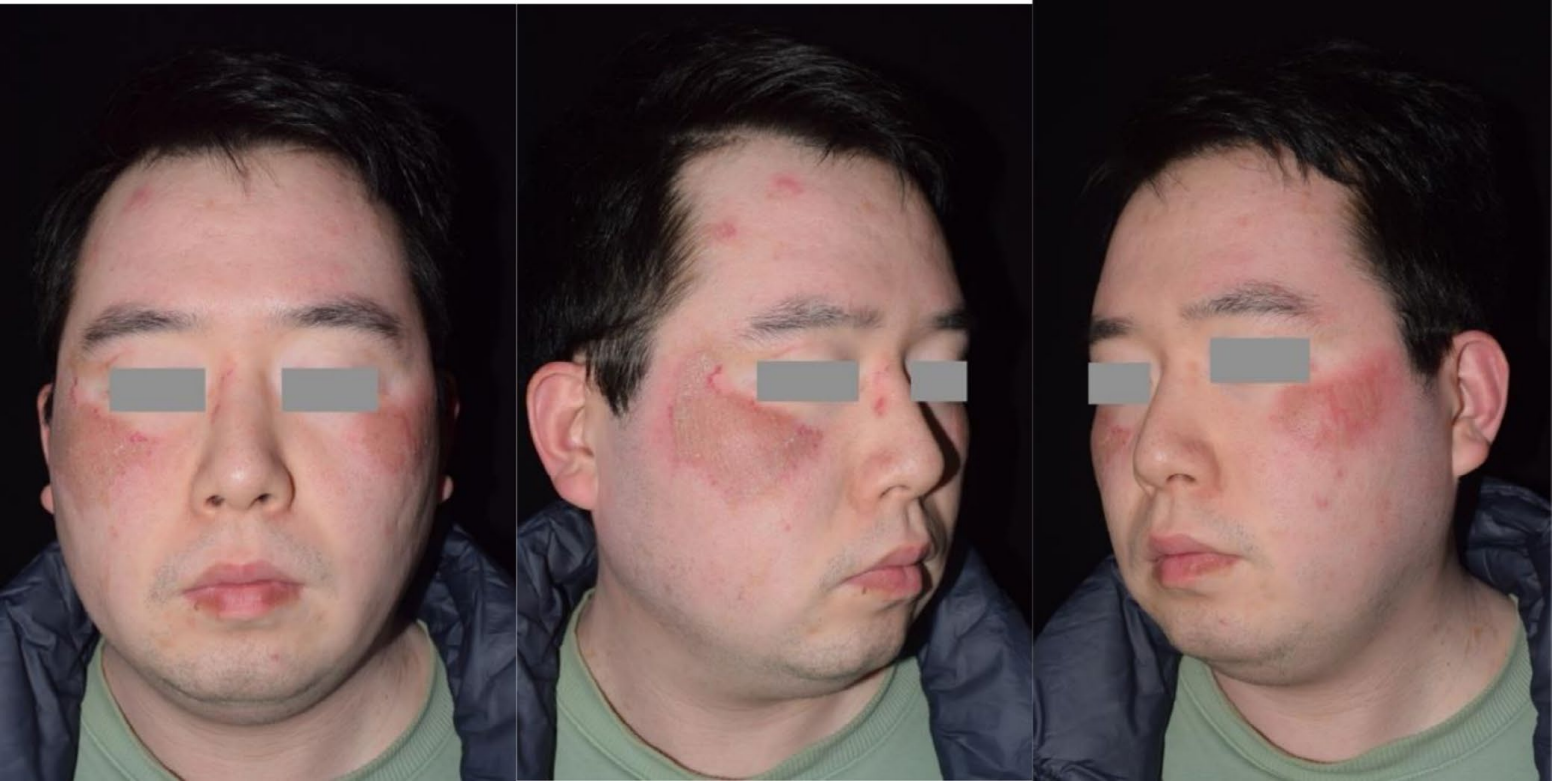
Immediate post‐laser image of Patient 1, demonstrating post‐treatment erythema and thin crust formation bilaterally.

Depigmentation therapy was initiated using a 532 nm picosecond laser (PicoWay, Candela, USA), the primary test spot targeting a 2 cm^2^ area on the left side of the face (3 mm, 0.8 J/cm^2^, 194 pulses). After 2 months, the test spot demonstrated acceptable therapeutic efficacy, and the patient underwent the bilateral facial treatment covering a 13 cm^2^ skin area (4 mm, 0.8–0.9 J/cm^2^, 1 Hz, 659 pulses). The treatment immediately induced erythema, mild edema, thin crust formation, and a transient burning sensation. These acute side effects were alleviated by short‐term application of hydrocortisone butyrate and resolved within approximately 1 week (Figure 2), followed by sequential application of 2% hydroquinone cream for 2 months, alongside strict photoprotection to prevent post‐inflammatory hyperpigmentation.

**FIGURE 3:**
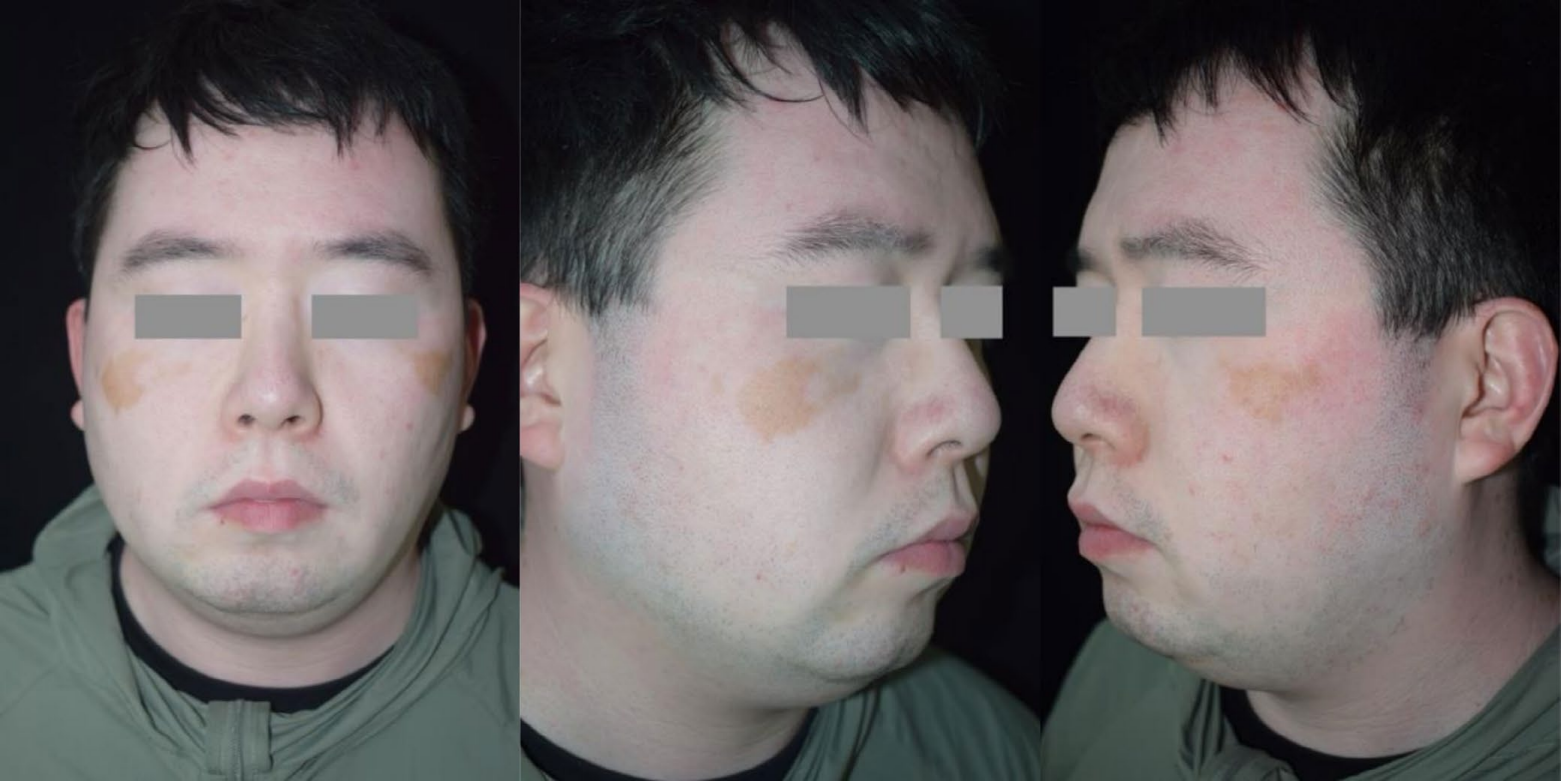
Six‐month post treatment image of Patient 1, showing nearly complete blending of depigmented areas with residual very slight hyperpigmented skin.

At the 6‐month follow‐up, significant blending of residual normal skin with surrounding depigmented areas was observed (Figure 3). The patient reported high satisfaction with the cosmetic outcome and remains under regular follow‐up.

### 2.2 Case 2

**FIGURE 4:**
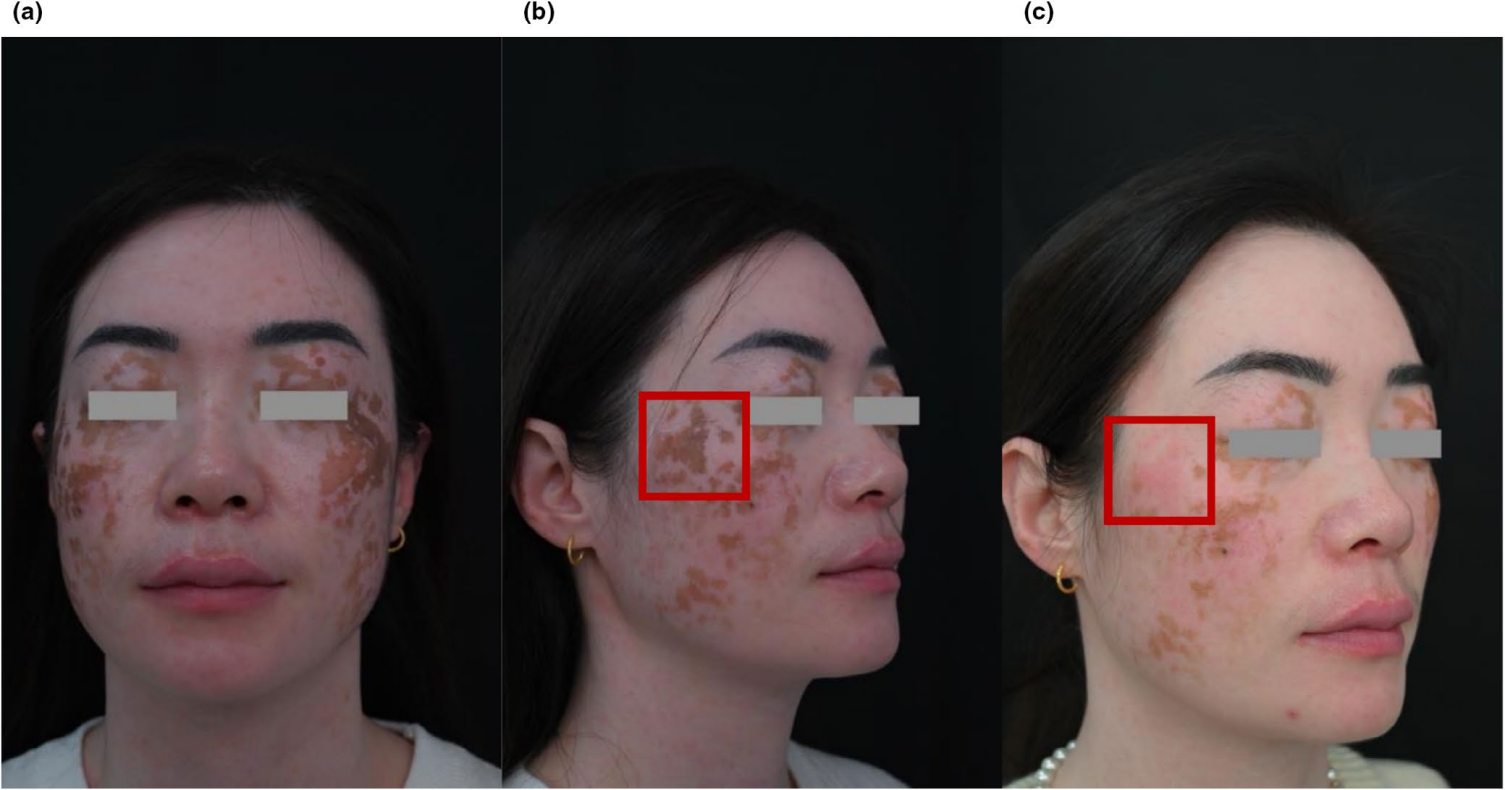
(a) Pre‐treatment image of Patient 2; (b) Targeted laser treatment of the right cheek (red box) during the first session; (c) 1‐month post treatment image of the treated area.

Since 7 years old, a 39‐year‐old female with vitiligo has had extensive depigmentation and rapidly progressed during pregnancy 10 years ago. While most of her skin has become depigmented, only residual scattered hyperpigmented macules on the face have been found cosmetically distressing. Clinical examination revealed near‐total skin depigmentation, with mottled brown macules on both cheeks and wrists (Figure 4).

**FIGURE 5:**
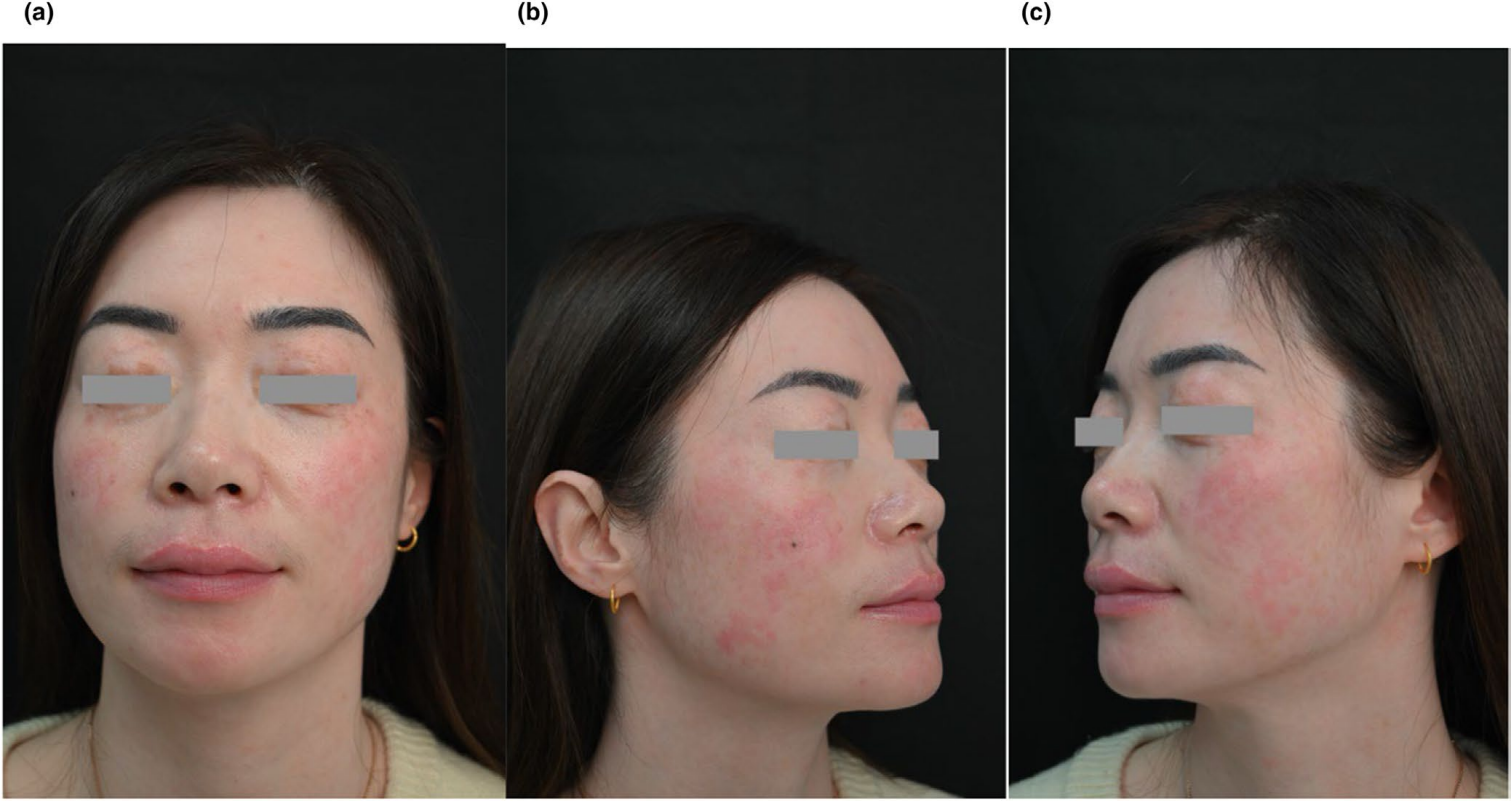
One‐month post treatment image of Patient 2, demonstrating near‐completeclearance of hyperpigmented macules following full‐face laser treatment.

The patient underwent two sessions of a 532 nm picosecond laser (PicoWay, Candela, USA) to address hyperpigmentation. In the first session, a 2 cm^2^ area was treated (3 mm, 0.9 J/cm^2^, 2 Hz, 111 pulses). One month later, full‐face treatment was performed (3 mm, 0.9 J/cm^2^, 2 Hz, 1275 pulses). The procedure was followed by 2% hydroquinone cream applied once or twice daily for 1 month. At the 1 month follow‐up after the second session, almost 100% clearance of facial hyperpigmentation was achieved. The patient observed significant aesthetic improvement (Figure 5).

## 3 Discussion

Depigmentation therapy has become an integral part of comprehensive vitiligo management, particularly for patients with extensive depigmentation and scattered residual pigmentation, with the goal of achieving a uniform skin tone. Several therapeutic approaches are currently available, each with distinct advantages and limitations regarding efficacy, safety, and patient tolerance.

Monobenzone and Monobenzyl ether of hydroquinone (MBEH), permanent depigmenting agents, are frequently employed in dermatological practice for pigmentary lesion removal. These compounds require prolonged application (≥ 10 months) yet demonstrate variable therapeutic outcomes, including non‐response and partial depigmentation. Treatment‐associated irritant erythema, pruritus, and edema may adversely impact patients' quality of life. High‐concentration trichloroacetic acid (TCA) serves as an alternative depigmenting agent, inducing milder localized burning sensations in the majority of patients. Ahmad Nofal et al. conducted a study on 50 patients with residual facial pigmentation using 100% trichloroacetic acid (TCA) administered biweekly (maximum 5 sessions, median 2 sessions). Results demonstrated > 90% pigmentation clearance in 80% of patients, 50%–90% improvement in 12%, and < 50% in 8%, confirming its high efficacy and cost‐effectiveness for refractory facial hyperpigmentation. This regimen demonstrated superior efficacy compared to prior studies utilizing lower TCA concentrations (25% or 50%) with tolerable adverse effects [1].

Cryotherapy emerges as another cost‐effective depigmentation modality. A clinical study comparing the dipstick method (three freeze cycles of 5–10 s/session) versus the open spray technique (single or double freeze–thaw cycles) demonstrated significantly fewer adverse events (*p* < 0.05) and superior patient satisfaction (*p* = 0.007), while maintaining comparable efficacy (77.5% depigmentation rate). Patients with darker skin exhibited enhanced therapeutic responsiveness. Standardized regimens (1–3 sessions at 4–6‐week intervals) induce durable depigmentation without scarring or long‐term complications [2].

Laser depigmentation therapy represents a highly efficient and safe approach. Q‐switched laser systems achieve precise depigmentation through selective photothermal disruption, utilizing pulse durations that correspond to the thermal relaxation time of cellular melanosomes. Commonly employed wavelengths include 694 nm ruby laser (QSRL), 755 nm alexandrite laser (QSAL), and 1064 nm Nd:YAG (QS Nd:YAG) lasers, which exhibit wavelength‐dependent specificity for pigment clearance across distinct skin depths. The QS Nd:YAG laser targets deeper dermal melanocytes due to enhanced tissue penetration, whereas the QSRL demonstrates superior epidermal basal layer pigment clearance efficacy. In previous studies comparing the Q‐switched Nd:YAG laser (1064/532 nm), cryotherapy, and chemical peels, the laser group achieved a 23.7% higher clearance rate (*p* < 0.05). This wavelength‐dependent efficacy necessitates individualized treatment selection based on Fitzpatrick skin phototype and anatomical lesion [3]. Clinical studies confirm 89.3% (95% confidence interval, CI: 82.1%–94.5%) residual pigment clearance in acral and facial vitiligo lesions using QSRL, with no severe complications such as scarring or dyschromia [4]. For refractory pigmentation, a single QS laser session achieves a median depigmentation rate of 76.8% (interquartile range, IQR: 54.2%–92.1%), with complete clearance attained in 81.4% of cases after five treatments [5]. However, pigment recurrence may occur months post‐treatment, particularly in facial and hand regions, necessitating annual maintenance laser sessions.

Picosecond lasers exhibit superior thermal relaxation characteristics compared to nanosecond systems, attributed to their ultrashort pulse durations (sub‐nanosecond regime) that confine energy deposition within melanosomal targets, thereby mitigating peri‐lesional thermal injury and improving side‐effect profiles. Nevertheless, existing evidence indicates insufficient melanocytic ablation during vitiligo depigmentation protocols employing picosecond lasers, potentially leading to suboptimal therapeutic outcomes.

Alvarez Martínez et al. demonstrated in a seven‐patient cohort with vitiligo that 532 nm picosecond laser therapy achieved complete depigmentation in four patients, near‐complete (≥ 90%) clearance in one, and therapeutic failure in two. Despite rigorous photoprotective protocols, two patients exhibited recurrence during summer. The regimen demonstrated favorable tolerability, with transient adverse events limited to erythema, crusting, vesiculation, and post‐inflammatory hyperpigmentation. These outcomes suggest comparatively reduced efficacy of picosecond lasers versus Q‐switched (QS) systems in vitiligo depigmentation [6]. Accordingly, both patients in this report were advised to implement strict sun protection in order to minimize the risk of post‐treatment recurrence or hyperpigmentation. A proposed pathomechanistic hypothesis posits that sub‐nanosecond pulse durations—while advantageous for confined photothermal litholysis of exogenous pigments (e.g., tattoo ink particles) and epidermal preservation through minimized thermal diffusion—may fail to generate critical cavitation thresholds (> 50 MPa transient pressure) required for complete melanocytic cytolysis, thereby limiting melanocyte ablation efficacy in depigmentation therapy.

Contrary to these findings, two Fitzpatrick type III patients with acrofacial vitiligo in this report achieved rapid optimal and consistent depigmentation after 1–2 sessions of 532 nm picosecond laser treatment, potentially linked to immediate endpoint reactions (frosted whitening, higher laser fluences than lentigo protocols). Post‐treatment cold compression and short‐term hydrocortisone butyrate application mitigated erythema and pruritus, followed by sequential 2% hydroquinone cream for 1–2 months yielding sustained outcomes without repigmentation at follow‐up.

Laser fluence optimization constitutes a critical determinant in achieving optimal therapeutic outcomes. In vitro investigations utilizing dynamic light scattering (DLS) and scanning electron microscopy (SEM) have established melanosomal disruption threshold fluences for picosecond lasers: 532 nm (0.95 J/cm^2^), 730 nm (2.25 J/cm^2^), 785 nm (2.75 J/cm^2^), and 1064 nm (6.50 J/cm^2^) [7]. These thresholds inform clinical parameter selection for picosecond laser‐mediated vitiligo depigmentation therapy. Furthermore, mechanistic studies of aberrant intracellular melanosome destruction may refine treatment protocols for recurrent pigmented lesions. Experimental data quantitatively correlate irradiation wavelength, incident fluence, and spot size with melanosome disruption at varying cutaneous depths.

In summary, wavelength selection in laser therapy operates through wavelength‐dependent melanosome disruption selectivity and pulse duration–thermal relaxation time (TRT) correlations. The 532 nm picosecond laser represents an efficacious and safe depigmentation modality. Distinct laser systems exhibit heterogeneous mechanisms and therapeutic profiles in vitiligo management, necessitating device selection based on individualized parameters including vitiligo subtype (segmental or non‐segmental), disease activity (stable or progressive), and anatomical site. Optimal fluence titration enhances cutaneous photo responsiveness, thereby potentiating depigmentation efficacy. Adjunctive post‐procedural care protocols combined with low‐concentration depigmenting agents (e.g., 2% hydroquinone) improve therapeutic maintenance and mitigate scarring or repigmentation risks.

## Author Contributions

Fang Wang and Juan Du contributed to the study conception and design, patient recruitment, and manuscript revision. Xinxin Li and Fang Wang drafted the initial version of the manuscript. Xiaolan Ding performed clinical assessments. Xuhai Yuan was responsible for patient follow‐up and clinical evaluation. All authors read and approved the final manuscript.

## Consent

Written informed consent was obtained from the patients for publication of the details of their medical information and any accompanying images.

## Conflicts of Interest

The authors declare no conflicts of interest.

## Data Availability

The data that support the findings of this study are available on request from the corresponding author. The data are not publicly available due to privacy or ethical restrictions.
